# Liquid Biopsy: Current advancements in clinical practice for bladder cancer

**DOI:** 10.1016/j.jlb.2025.100310

**Published:** 2025-07-08

**Authors:** Felice Crocetto, Ugo Amicuzi, Michele Musone, Marco Magliocchetti, Dario Di Lieto, Simone Tammaro, Antonio Luigi Pastore, Andrea Fuschi, Roberto Falabella, Matteo Ferro, Roberto Bianchi, Marco Finati, Gian Maria Busetto, Giuseppe Lucarelli, Francesco Del giudice, Vincenzo Francesco Caputo, Raffaele Balsamo, Daniela Terracciano

**Affiliations:** a Department of Neurosciences, Reproductive Sciences and Odontostomatology, University of Naples “Federico II”, Naples, Italy; b Urology Unit, Department of Medico-Surgical Sciences and Biotechnologies, Faculty of Pharmacy and Medicine, Sapienza University of Rome, 04100, Latina, Italy; c Urology Unit, San Carlo Hospital, Via Potito Petrone, 85100, Potenza, Italy; d Unit of Urology, Department of Health Science, University of Milan, ASST Santi Paolo e Carlo, 20172, Milan, Italy; e Department of Urology and Renal Transplantation, University of Foggia, 71122, Foggia, Italy; f Urology and Kidney Transplantation Unit, Department of Precision and Regenerative Medicine and Ionian Area-Urology, University of Bari "Aldo Moro", 70124 Bari, Italy; g 5SSD Urologia Clinicizzata, IRCCS Istituto Tumori "Giovanni Paolo II", 70124 Bari, Italy; h Department of Maternal Infant and Urological Sciences, Sapienza University of Rome, Policlinico Umberto 1 Hospital, Rome, Italy; i Urology Unit, AORN Ospedali dei Colli, Monaldi Hospital, 80131, Naples, Italy; j Department of Translational Medical Sciences, University of Naples “Federico II”, Italy

**Keywords:** Liquid, Biopsy, Bladder cancer, Circulating, Biomarkers

## Abstract

Bladder cancer is the ninth most common malignancy worldwide, with two clinically distinct forms: non-muscle-invasive disease, characterized by high recurrence and excellent long-term survival, and muscle-invasive disease, associated with poorer outcomes. Current surveillance—cystoscopy and urine cytology—offers high specificity but is invasive, costly, and insensitive to low-grade tumors, underscoring the need for reliable, non-invasive biomarkers. Liquid biopsy approaches in urine and blood have demonstrated promise for real-time assessment of tumor burden, molecular heterogeneity, and early recurrence. Circulating tumor DNA (ctDNA) assays detect tumor-derived genetic and epigenetic alterations, enabling dynamic monitoring of minimal residual disease and treatment response. Methylation-based tests and CpG-targeted sequencing in urine achieve high diagnostic accuracy, potentially reducing dependence on cystoscopy. Molecular classification of bladder tumors into luminal and basal subtypes has refined therapeutic strategies: FGFR inhibitors for luminal-papillary tumors, EGFR-targeted and chemotherapy approaches for basal/squamous cases, and immune-checkpoint inhibitors guided by immune-infiltration profiles. Integration of artificial intelligence with multi-omic liquid biopsy data further enhances predictive modeling for recurrence, treatment response, and minimal residual disease detection. Despite these advances, clinical implementation faces challenges including pre-analytical variability, lack of standardized assays, limited prospective validation, and unclear cost-effectiveness. Harmonized protocols, large multicenter trials, and health-economic evaluations are essential to translate liquid biopsy technologies into routine practice. Future integration with advanced imaging, tissue biopsy, and digital pathology—supported by multidisciplinary collaboration and formal guideline endorsement—holds the potential to personalize bladder cancer management, reduce invasive procedures, and improve patient outcomes.

## 1 Introduction

**Fig. 1:**
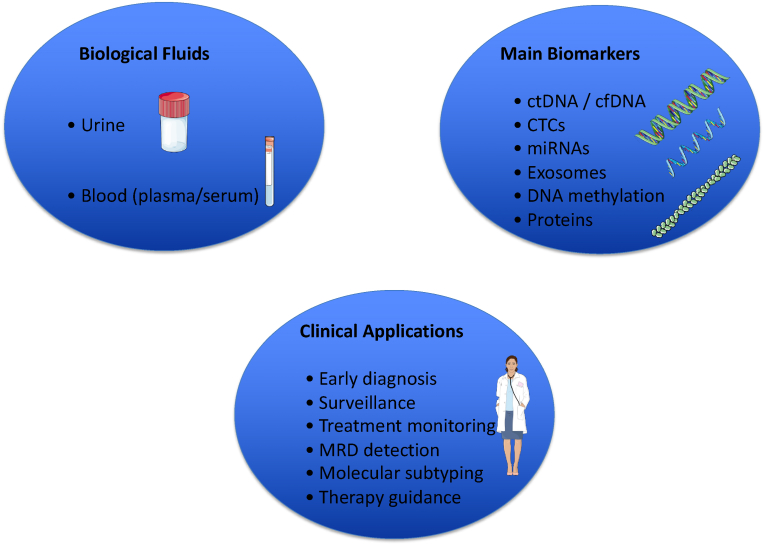
Overview of Liquid Biopsy Applications in Bladder Cancer. Parts of the figure were drawn using pictures from Server Medical Art. Servier Medical Art by Servier is licensed under a Creative Commons Attribution 3.0 Unported License.

Bladder cancer is the ninth most common malignancy worldwide, accounting for approximately 550,000 new cases and 165,000 deaths annually, with a markedly higher incidence in men (age-standardized rate of 9 per 100,000) than in women (2.2 per 100,000) [1]. The disease is broadly classified into NMIBC, which comprises 70–80 % of newly diagnosed cases and carries a 5-year survival rate of over 95 % for carcinoma in situ and Ta lesions, and muscle-invasive bladder cancer (MIBC), which has substantially lower survival rates (approximately 34.9 % for T3 lesions) [1]. Major risk factors include tobacco smoking and occupational exposure to aromatic amines, contributing to the substantial morbidity and mortality associated with BC [2] (see Table 1, Fig. 1).

**Table 1:** Main clinical trials on liquid biopsy in bladder cancer.

Trial/Study	Biomarker Type	Population	Clinical Endpoint	Reference
Birkenkamp-Demtröder et al., 2018	ctDNA (plasma)	Advanced bladder cancer	MRD detection and relapse monitoring	Eur Urol 2018; 73 (4):535-540
Christensen et al., 2019	cfDNA (plasma)	Post-cystectomy surveillance	Early detection of relapse	J Clin Oncol 2019; 37 (18):1547-1557
Nordic LS-URO Trial	Urine ctDNA (home-based)	Lynch syndrome carriers	Screening and early detection	ClinicalTrials.gov NCT05204552
Vandekerkhove et al., 2017	ctDNA (metastatic)	Metastatic urothelial carcinoma	Mutation profiling and therapy guidance	Clin Cancer Res 2017; 23 (21):6487-6497
Shen et al., 2018	cfDNA methylomes (plasma)	Bladder and other cancers	Stage-agnostic detection	Nature 2018; 563:579–583
Ward et al., 2016	CpG panel (urine)	NMIBC surveillance	Diagnostic performance (AUC 0.97)	PLoS One 2016; 11 (2):e0149756
Beukers et al., 2017	FGFR3, TERT, OTX1 mutations (urine)	Bladder cancer surveillance	Mutation detection and recurrence risk	J Urol 2017; 197 (6):1410-1418

Current clinical practice relies heavily on cystoscopy and urine cytology for diagnosis and surveillance. Urine cytology offers high specificity (∼98 %) but low overall sensitivity (∼38 %), improving only in high-grade lesions (>60 %) [3]. Cystoscopy achieves sensitivities of 85–90 % for papillary tumors and 65–70 % for carcinoma in situ but is invasive, costly, and prone to inter-observer variability in tumour staging and grading [3].

NMIBC is notorious for its high recurrence rate (50–70 %) and risk of progression (10–15 %), necessitating frequent follow-up with cystoscopy every 3–6 months and urine cytology, which makes BC the most expensive cancer from diagnosis to death, with an estimated per-patient cost of USD 187,000 in the United States and annual expenditures exceeding USD 4 billion globally [1]. These challenges highlight the pressing need for reliable, non-invasive biomarkers to complement or even replace existing modalities [4,5].

Liquid biopsy refers to the sampling of biological fluids - such as blood, plasma and urine - to detect tumour-derived material, including CTCs, ctDNA, mRNAs, microRNAs, long non-coding RNAs, proteins, metabolites and extracellular vesicles (exosomes) [6]. This approach enables dynamic, real-time assessment of tumour burden, molecular heterogeneity, minimal residual disease and emerging resistance mechanisms without invasive procedures. In bladder cancer, several studies have shown that ctDNA and CTC analyses can predict prognosis, monitor treatment response and detect early recurrence, offering a transformative potential for personalized patient management [7].

## 2 Materials and methods

A systematic literature search was performed in PubMed, Embase and Web of Science from database inception through April 2025 using the terms “bladder cancer” AND (“liquid biopsy” OR “ctDNA” OR “circulating tumor DNA” OR “circulating tumor cells” OR “exosomes” OR “urinary biomarkers”). After deduplication, titles and abstracts were screened independently by two reviewers against prespecified inclusion criteria—original, peer-reviewed studies in English evaluating blood- or urine-based liquid biopsy markers in bladder cancer—and excluding case reports, conference abstracts and non-English articles. Full texts of potentially eligible papers were then assessed; data on study design, patient cohort, assay type, target analyte and diagnostic or prognostic performance metrics were extracted using a standardized form. Discrepancies at each stage were resolved by consensus. Extracted results were qualitatively synthesized according to biomarker category (e.g., ctDNA, CTCs, miRNAs, exosomal cargo) to provide a structured overview of current evidence and identify areas for future research.

## 3 Biomarkers in bladder cancer

Urine cytology remains the most widely used non-invasive test for bladder cancer detection, with an overall sensitivity of approximately 37 % and specificity of 95 % for all stages; sensitivity drops dramatically for low-grade tumors (<20 %), underscoring the need for more sensitive assays [8]. Protein-based urinary assays were among the first extensions beyond cytology. Liquid biopsy refers to the analysis of tumor-derived material present in biological fluids, most notably urine, blood, plasma, and serum. In bladder cancer, urine represents an especially informative matrix due to the direct contact between the urothelial tumor and the urinary tract, enabling the non-invasive detection of exfoliated tumor cells, nucleic acids, proteins, and extracellular vesicles. Meanwhile, blood-based specimens, such as plasma and serum, offer valuable systemic insights, particularly for monitoring circulating tumor DNA (ctDNA) and circulating tumor cells (CTCs) in advanced or metastatic stages. The combined use of both urine and blood thus allows for comprehensive molecular profiling, real-time disease monitoring, and early detection of recurrence, positioning liquid biopsy as a powerful tool across the entire spectrum of bladder cancer management. The NMP22 BladderChek® immunoassay, which detects a nuclear matrix-associated protein released by apoptotic urothelial cells, demonstrated a pooled sensitivity of 56 % (95 % CI, 52–59 %) and specificity of 88 % (95 % CI, 87–89 %) across 19 quantitative studies (n = 5197), with a diagnostic odds ratio of 9.29 and AUC of 0.83 [9]. Similarly, BTA STAT and BTA TRAK assays targeting complement factor-H related proteins exhibit higher sensitivity than cytology (≈64–75 %) but lower specificity (≈74–77 %) in meta-analyses, limiting their standalone utility due to false positives in benign conditions such as inflammation or stones [10]. Fluorescence in situ hybridization (UroVysion®) detects aneuploidy of chromosomes 3, 7, 17 and 9p21 deletion in urinary cells. A meta-analysis of 14 studies (n = 2477) reported pooled sensitivity of 72 % (69–75 %) and specificity of 83 % (82–85 %), outperforming cytology in sensitivity (42 %) but with somewhat lower specificity (96 %) [11]. More recently, methylation-based PCR assays have shown excellent performance. The Bladder EpiCheck™ test analyzes a panel of 15 DNA methylation markers in urine and, in a multicenter validation of 222 NMIBC surveillance samples, achieved 90 % sensitivity, 83 % specificity and 97 % negative predictive value [12]. A next-generation sequencing approach, UroMark, targets 150 CpG loci in urinary sediment DNA. In proof-of-concept and validation cohorts (n = 274), UroMark attained an AUC of 0.97, sensitivity of 98 %, specificity of 97 % and NPV of 97 %, suggesting potential to replace cystoscopy for primary detection [13]. In addition to urine cytology and protein-based markers, significant advances have been made in the application of nucleic acid biomarkers for bladder cancer. MicroRNAs (miRNAs), particularly those identified in urine and serum, have shown diagnostic and prognostic potential, with several studies reporting robust miRNA signatures capable of distinguishing malignant from benign conditions and stratifying disease aggressiveness. Similarly, cell-free DNA (cfDNA) and ctDNA derived from plasma or urine enable dynamic, non-invasive assessment of tumor burden, clonal evolution, and minimal residual disease. Circulating tumor cells (CTCs), although less commonly detected in early-stage disease, have been associated with progression-free and overall survival in metastatic urothelial carcinoma. These biomarkers, individually and in combination, hold promise for guiding clinical decisions across multiple stages of disease.

### 3.1 Urinary miRNA panels

MiRNAs in urine supernatant offer another non-invasive layer of tumour-derived information. Several urinary microRNA (miRNA) signatures have been proposed for bladder cancer detection and risk stratification. In addition to the seven-miRNA panel (miR-221–3p, miR-93–5p, miR-362–3p, miR-191–5p, miR-200c-3p, miR-192–5p, miR-21–5p), which showed 75 % sensitivity and 70 % specificity in distinguishing cancer cases from controls [14], other studies have validated alternative panels with comparable or superior performance. For example, miR-126, miR-182, and miR-96 have been associated with tumor grade and recurrence risk, while reduced urinary levels of tumor-suppressive miRNAs, such as miR-145 and miR-143, have been linked to disease progression [50]. Notably, several of these miRNA signatures exhibit high negative predictive value, suggesting utility in surveillance protocols to reduce unnecessary cystoscopy. As a result, urinary miRNAs are emerging not only as diagnostic tools but also as potential prognostic biomarkers for clinical outcome prediction.

### 3.2 Blood-based ctDNA and CTCs

CtDNA in plasma provides a complementary, blood-based liquid biopsy. ctDNA and circulating tumour cells (CTCs) have been shown to predict prognosis, monitor minimal residual disease and capture emerging resistance, with clinical trials underway to evaluate ctDNA-guided adjuvant therapy in MIBC [15,16]. Although detection rates vary by stage - lower in NMIBC and higher in metastatic disease - advancements in assay sensitivity and fragmentomic analyses are rapidly improving ctDNA's diagnostic potential. Recent studies have reinforced the clinical utility of blood-based ctDNA in bladder cancer across multiple disease stages. Detection rates correlate with tumor burden, with higher ctDNA levels observed in muscle-invasive and metastatic disease, while ultrasensitive assays are increasingly able to capture ctDNA even in non-muscle-invasive settings. Longitudinal monitoring of ctDNA during and after treatment enables early detection of minimal residual disease (MRD) and relapse, often preceding radiographic evidence by several months. Furthermore, ctDNA dynamics have been successfully integrated into adjuvant therapy trials, where clearance of ctDNA post-chemotherapy or surgery is associated with improved progression-free and overall survival. These findings support the inclusion of plasma ctDNA as a minimally invasive biomarker to guide therapeutic decision-making and assess real-time treatment efficacy [17]. CTCs represent a valuable, though less commonly utilized, component of liquid biopsy in bladder cancer. Their presence in peripheral blood has been associated with advanced disease stage, shorter progression-free survival, and poor overall prognosis. Advances in enrichment and detection technologies, such as microfluidic platforms and immunomagnetic separation, have improved CTC yield and characterization. Importantly, CTC analysis offers dynamic insight into treatment response and the emergence of drug resistance. Molecular profiling of isolated CTCs allows the identification of actionable mutations, epithelial-to-mesenchymal transition markers, and expression of therapeutic targets such as PD-L1. While CTC enumeration has not yet been incorporated into routine clinical workflows, their potential for real-time monitoring and resistance tracking underscores their growing relevance in precision oncology and supports ongoing efforts toward standardization and validation in prospective trials [18,19].

### 3.3 Prognostic biomarkers

Prognostic biomarkers inform on the likely course of disease, including risk of recurrence and overall survival.

### 3.4 FGFR3 (fibroblast growth factor receptor 3) mutations

Recent prospective evidence has reinforced the prognostic value of *FGFR3* and telomerase reverse transcriptase (*TERT* promoter mutations in bladder cancer. Activating mutations in *FGFR3* are commonly observed in NMIBC, particularly in low-grade, low-stage tumors, and are associated with favorable outcomes, including reduced risk of progression and prolonged recurrence-free survival. Conversely, somatic mutations in the *TERT* promoter, especially the −124C > T and −146C > T hotspots, are highly prevalent across all stages and grades of bladder tumors and have emerged as strong prognostic indicators. In a large multi-institutional cohort, *TERT* promoter mutations were independently associated with increased risk of disease recurrence (hazard ratio [HR] 1.85; 95 % CI, 1.11–3.08) and worse overall survival (HR 2.19; 95 % CI, 1.02–4.70), even in the absence of other high-risk features. These findings support the incorporation of molecular profiling into routine risk stratification and follow-up protocols [20,21].

### 3.5 *TERT* promoter mutations

Somatic mutations in the *TERT* promoter (−124C > T and −146C > T) are detected in ∼65 % of bladder tumors across all stages. In a cohort of 327 patients, *TERT* promoter mutations were associated with increased risk of disease recurrence (HR 1.85; 95 % CI, 1.11–3.08) and worse overall survival (HR 2.19; 95 % CI, 1.02–4.70) in the absence of a modifying Ets-binding polymorphism [22,23].

### 3.6 Predictive biomarkers

Predictive biomarkers in NMIBC have focused on urinary cytokines, tissue markers and immune phenotypes to identify patients most likely to benefit from intravesical BCG. Among urinary cytokines, IL-2 has emerged as the most extensively studied predictor: higher IL-2 levels measured during BCG induction correlate strongly with improved recurrence-free survival and reduced recurrence rates [24]. Similarly, elevated urinary IL-8 - both during induction and maintenance phases - has been associated with better treatment outcomes and longer recurrence-free intervals, while increased TNF-α concentrations following BCG instillation also predict favorable therapeutic responses in NMIBC [25]. Tissue-based analyses have evaluated the expression of cell-cycle regulators in TURBT specimens: abnormal p53 and pRb status correlates with diminished BCG efficacy, particularly in high-risk T1G3 tumors, suggesting that perturbations in these pathways may underlie resistance to intravesical therapy [26]. More recently, multidimensional pre-treatment immunoprofiling has been explored to distinguish “inflamed” from “desert” tumour immune landscapes. Early data indicate that an inflamed phenotype - with robust T-cell infiltration and upregulated immune checkpoints - predicts a higher likelihood of durable response to BCG, whereas immune-desert tumors may warrant early consideration of alternative immunotherapies [27]. The clinical relevance of predictive biomarkers in NMIBC lies in their potential to optimize patient selection for intravesical Bacillus Calmette-Guérin (BCG) therapy and improve treatment outcomes. Urinary cytokines such as interleukin-2 (IL-2), interleukin-8 (IL-8), and tumor necrosis factor-alpha (TNF-α) provide dynamic, non-invasive indicators of immune activation in response to BCG. Elevated IL-2 levels during induction have been associated with enhanced recurrence-free survival and lower recurrence rates, suggesting that IL-2 may serve as a surrogate of effective antitumor immunity. Likewise, higher urinary IL-8 and TNF-α levels during both induction and maintenance phases correlate with improved therapeutic response, offering a practical tool for early prediction of BCG efficacy. Tissue-based biomarkers, such as aberrant expression of p53 and pRb in transurethral resection specimens, have been linked to reduced BCG responsiveness, particularly in high-risk T1G3 tumors, underscoring the importance of genomic integrity in treatment success. Additionally, pre-treatment immunoprofiling distinguishing “inflamed” from “immune-desert” tumors has emerged as a powerful approach for predicting BCG response. Tumors with a preexisting T-cell–rich microenvironment and checkpoint upregulation are more likely to exhibit durable benefit from immunotherapy, while immune-desert phenotypes may prompt early escalation to alternative or combination therapies. Together, these biomarkers offer actionable insights to guide personalized management of NMIBC and reduce unnecessary exposure to ineffective treatment.

## 4 Molecular classifications in bladder cancer

### 4.1 Luminal and basal subtypes

Initial transcriptomic analyses by The Cancer Genome Atlas (TCGA) defined five discrete MIBC subtypes - luminal-papillary, luminal-infiltrated, luminal, basal-squamous and neuronal - based on unsupervised mRNA clustering, highlighting the dichotomy between urothelial differentiation and basal-like programs [28,29]. Kamoun and colleagues then compared six independent classification schemes across 1750 MIBC samples to propose a consensus taxonomy comprising six classes: luminal-papillary (LumP), luminal-non-specified (LumNS), luminal-unstable (LumU), stroma-rich, basal/squamous (Ba/Sq) and neuroendocrine-like (Ne-like) [30]. Luminal-papillary tumors are marked by high expression of urothelial differentiation markers - such as KRT20, GATA3, UPK1A and UPK2 - and frequent activating FGFR3 mutations or fusions, reflecting their origin from terminally differentiated umbrella cells [31,32]. In contrast, basal/squamous tumors express keratins 5/6 and CD44, share transcriptional features with basal-like breast and squamous head and neck cancers, and exhibit elevated EGFR signaling, indicative of a more progenitor-like, aggressive phenotype [30,31]. Luminal-infiltrated and stroma-rich subtypes both display significant non-tumor components - immune cells in the former and fibroblasts/extracellular matrix in the latter - whereas the LumU class is characterized by upregulated cell-cycle genes and genomic instability [33,34].

### 4.2 Implications for personalized medicine

The distinct molecular features of these subtypes have direct therapeutic implications. Luminal-papillary tumors, enriched for *FGFR3* alterations, are sensitive to FGFR inhibitors such as erdafitinib, which has shown clinical benefit in *FGFR3*-mutant urothelial carcinoma [35,36]. Ba/Sq tumors, despite their generally poorer prognosis, demonstrate higher response rates to cisplatin-based chemotherapy and may benefit from EGFR-targeted approaches given their receptor expression profile [[33], [37]]. Immune-infiltrated and basal/squamous subtypes also overexpress checkpoints such as PD-L1 and CTLA-4, suggesting they are prime candidates for immune-checkpoint blockade [38]. Molecular stratification thus provides a robust framework for matching therapies to tumor biology; however, prospective validation in clinical trials and standardized, reproducible assays are essential before widespread implementation in routine practice [39].

## 5 Emerging technologies and biomarker discovery

Emerging liquid biopsy modalities - particularly circulating tumor DNA (ctDNA) and urinary exosomes - are revolutionizing biomarker discovery in bladder cancer by enabling non-invasive, real-time molecular profiling that captures intra-tumoral heterogeneity and dynamic changes under therapy [40].

### 5.1 Circulating tumor DNA

In bladder cancer, ctDNA analysis has emerged as a powerful tool for detecting tumor-derived genetic and epigenetic alterations in plasma and urine. Urine tumor DNA (utDNA) assays have demonstrated impressive diagnostic performance, with one meta-analysis reporting a sensitivity of 91 % and specificity of 96 % for non-invasive detection, outperforming cystoscopy and cytology for tumor detection and surveillance [41]. Targeted sequencing of ctDNA methylation patterns further outperforms fluorescence in situ hybridization and mutation-based assays, enabling earlier detection and prognostication in both NMIBC and MIBC cohorts [42]. Longitudinal assessment of ctDNA provides prognostic and minimal residual disease (MRD) information: detectable ctDNA prior to radical cystectomy predicts poor recurrence-free survival (HR 4.5; 95 % CI 1.0–19.0), nodal involvement (OR 5.4; 95 % CI 1.9–18.2) and locally advanced disease (OR 3.6; 95 % CI 1.5–9.0) [39]. Whole-genome sequencing-based ctDNA detection extends lead time by a median of 131 days over radiographic imaging, identifying recurrence with 91 % sensitivity and 92 % specificity post-cystectomy [40]. In advanced urothelial carcinoma, ctDNA dynamics correlate with therapeutic response: post-neoadjuvant chemotherapy ctDNA clearance is independently associated with improved outcomes, while persistent ctDNA after cystectomy predicts metastatic relapse with 94 % sensitivity and 98 % specificity [16]. Despite these successes, challenges remain, including the lower abundance of ctDNA in early-stage NMIBC, the need for standardized ultra-sensitive assays such as CAPP-Seq with integrated error-suppression strategies, and the technical complexity of large-scale sequencing pipelines [43]. Additionally, methylomic and copy-number analyses of urinary cfDNA enable stage-agnostic tumor detection without prior knowledge of specific aberrations, further broadening ctDNA's diagnostic reach [44].

### 5.2 Urinary exosomes and extracellular vesicles

Extracellular vesicles, particularly exosomes, have gained attention as carriers of tumor-derived nucleic acids and proteins in bladder cancer. Exosomes isolated from urine - a biofluid in direct contact with urothelial tumors - can be purified via differential centrifugation or polymer-based precipitation, and contain microRNAs, mRNAs, long non-coding RNAs and protein cargos that mirror the tumor's molecular landscape [45]. Machine learning applied to urinary exosome miRNA expression has demonstrated diagnostic accuracy: integration of exosomal miRNA profiles with clinical data achieved an ROC of 0.85 and F1 score of 0.79 in distinguishing bladder cancer from controls [46]. Several reviews have underscored the functional roles of exosomal cargo in tumorigenesis - promoting angiogenesis, invasion and immune modulation - and their utility as biomarkers for both NMIBC and MIBC [47,48]. A meta-analysis of urinary exosome diagnostic studies across urological tumors reported pooled sensitivity and specificity of 83 % and 88 %, with an AUC of 0.92, affirming exosomes as a robust non-invasive tool for early detection and surveillance [49]. However, lack of standardized isolation and normalization procedures, as well as pre-analytical variables such as urine collection and storage, pose significant barriers to clinical translation and require harmonization in future studies [50].

### 5.3 Other recent technologies

Narrow Band Imaging (NBI) and blue-light cystoscopy with Hexvix (HAL-BLC) have emerged as advanced diagnostic modalities aimed at enhancing detection and management of NMIBC, each with distinct mechanisms, advantages, limitations, and cost implications [51,52]. NBI employs specific blue (415 nm) and green (540 nm) wavelengths to accentuate mucosal vascular patterns, enabling improved visualization of superficial lesions without the need for exogenous dyes or changes to standard cystoscopic setup [53]. Multiple studies have demonstrated that NBI cystoscopy significantly increases sensitivity for tumor detection compared to conventional white-light cystoscopy (WLC) - with sensitivities reported up to 96 % versus 73 % for WLC - although this gain in sensitivity is offset by a modest reduction in specificity and an elevated false-positive rate, particularly in inflammatory or benign lesions [54,55]. A recent meta-analysis confirmed that NBI improves per-patient and per-lesion detection rates of NMIBC and carcinoma in situ (CIS), and is associated with reduced recurrence rates, with one analysis reporting a 37 % lower likelihood of recurrence over 12–35 months [56,57]. Importantly, because NBI is activated by a simple switch on compatible endoscopes, it avoids consumable costs related to dye instillation, although the initial investment in NBI-capable equipment may be substantial [58]. By contrast, HAL-BLC requires intravesical instillation of hexaminolevulinate hydrochloride (Hexvix/Cysview) prior to cystoscopy to induce selective fluorescence within malignant urothelium under blue light excitation [59]. Clinical trials have shown that HAL-BLC identifies additional tumors missed on WLC, increasing detection rates of papillary lesions by 12–20 % and CIS by up to 43 %, thereby facilitating more complete tumor resection and improved risk stratification [52,60]. HAL-BLC introduces additional procedural steps, including intravesical instillation of Hexvix and a required 1-h dwell time prior to cystoscopic examination, which may impact scheduling and patient convenience [52]. It also carries an incremental cost per procedure that varies by healthcare setting; for example, adoption in a large French public hospital model was estimated to increase per-procedure costs by €269 (∼10 %), while a more targeted implementation in a smaller private unit yielded a €133 increase (∼5 %) [61]. Economic analyses from Sweden and Canada have suggested that, over one year and five years respectively, HAL-BLC may be cost-saving or cost-neutral due to decreases in recurrence and associated treatments, with reported savings of €73 per patient in Sweden and amortized costs of 1236−1236−1372 per patient in Canadian provinces alongside substantial reductions in recurrence burden [15,62]. However, budgetary concerns, limitations of clinical trial-derived recurrence data, and the need for real-world cost-effectiveness evaluation underscore the importance of further studies across diverse practice environments [49]. Meanwhile, use of circulating miRNAs in blood samples has also gained popularity. Blood-based tests provide the advantage of systematic biomarker detection, especially beneficial with high-grade or muscle-invasive tumors. The levels of serum miR-21 and miR-155, have correlated with the disease's stage and prognosis. Additional notable candidates include miR-192, miR-200c, and miR-214. These circulating miRNAs can also become a way to monitor treatment responses, particularly in those undergoing immunotherapy or BCG treatment and could help reveal any initial recurrence indicators [52].

## 6 Clinical implementation and challenges

### 6.1 Integration into clinical practice

Despite U.S. Food and Drug Administration clearance for several urine tests (e.g., NMP22, UroVysion), international clinical guidelines continue to recommend cystoscopy plus cytology as the gold standard for diagnosis and surveillance. A consensus review of urinary biomarkers concluded that, while assays can complement surveillance in high-risk patients, they are not yet reliable enough to replace cystoscopy or cytology in routine practice [52]. The 2024 European Association of Urology Guidelines on NMIBC similarly emphasize cystoscopic evaluation and consider urinary markers only in select scenarios - such as reducing cystoscopy frequency for very low-risk patients - underscoring their limited current role [63]. Early-phase studies in liquid biopsy (e.g., ctDNA panels, exosomal miRNA signatures) illustrate technical feasibility and potential for monitoring minimal residual disease, but clinical implementation remains confined to research settings where real-time molecular monitoring informs trial design rather than standard care [64].

### 6.2 Pre-analytical and analytical variability

Urine and blood specimen handling critically impact biomarker measurements. Variables such as time and method of collection (first-morning vs. random urine), addition of protease or nuclease inhibitors, centrifugation protocols, storage temperature, and shipping durations can alter protein, nucleic acid and extracellular vesicle yields and quality. Standardization efforts - including adherence to International Society for Extracellular Vesicles (ISEV) guidelines and use of centralized reporting via EV-TRACK - are essential to reduce inter-laboratory variability [65]. In exosome-based assays, lack of consensus on urine stabilizers, THP (Tamm-Horsfall protein) inhibitors, storage times (ideally ≤4 h), and separation methods (ultracentrifugation vs. immunocapture vs. microfluidics) impairs reproducibility and comparability across studies [9].

### 6.3 Clinical validation and cohort heterogeneity

Most biomarker studies to date involve small patient numbers, single-center cohorts and retrospective designs, limiting statistical power and generalizability. Heterogeneity in tumour stage, grade and prior treatments further confounds interpretation and validation of thresholds for positive calls. Without large, prospective, multi-institutional trials employing uniform protocols, the clinical validity and utility of candidate biomarkers - and their impact on patient outcomes - remain uncertain [35].

**Cost, Reimbursement and Health-Economics** High-throughput sequencing panels and advanced exosome platforms incur substantial upfront costs, specialized equipment and bioinformatics expertise. Although a review across cancer types found that liquid biopsy technologies were cost-effective in approximately 75 % of economic evaluations, more disease-specific analyses are needed for bladder cancer [16]. Protein-based tests like NMP22 offer lower per-test costs and demonstrated favorable cost-effectiveness in high-risk hematuria populations [66]. Payer coverage is increasing for ctDNA assays in oncology, but reimbursement policies remain variable across jurisdictions and biomarker types, limiting broad clinical adoption [41]. Continued demonstration of cost-utility in real-world settings - alongside streamlined, lower-cost workflows - will be critical to secure health technology assessment approval and integrate liquid biopsy into standard care pathways [67]. Through harmonized pre-analytical protocols, rigorous prospective validation and alignment with economic and regulatory frameworks, liquid biopsy biomarkers have the potential to augment or, in selected scenarios, reduce the frequency of invasive procedures in bladder cancer management. Addressing these challenges is essential to translate promising assays from bench to bedside.

## 7 Future perspectives

### 7.1 Advancements in precision oncology

Recent studies demonstrate that longitudinal ctDNA monitoring can identify minimal residual disease (MRD) and predict relapse weeks or months before imaging, enabling tailored adjuvant interventions. In metastatic urothelial carcinoma, changes in ctDNA allele fractions after neoadjuvant chemotherapy correlated strongly with pathological response and survival outcomes, underscoring its role in real-time treatment adaptation [48]. The Nordic LS-URO prospective trial is evaluating home-based urine ctDNA screening for Lynch syndrome carriers to trigger early urologic evaluation, exemplifying precision interception in hereditary BCa risk populations [54]. Multi-omic liquid biopsies that integrate ctDNA mutations, methylation profiles and fragmentomics are under development and have achieved >90 % sensitivity for NMIBC detection in early studies, paving the way for non-invasive disease stratification and surveillance [55].

### 7.2 Role of artificial intelligence in biomarker analysis

Machine-learning algorithms are being trained on multi-parametric liquid biopsy datasets - combining urinary miRNA panels, exosomal SERS signatures and clinical variables - to improve diagnostic accuracy and reduce false positives. A recent prospective study applied Random Forest, SVM and XGBoost classifiers to urinary exosome miRNA plus demographic and laboratory data, achieving >95 % accuracy for BCa detection, demonstrating the power of AI to refine biomarker panels [37,68]. Broad reviews of AI methodologies in bladder cancer emphasize the transition from historical nomograms to deep-learning models that can mine high-dimensional omics and imaging data to predict recurrence and BCG response [38]. Advanced AI-driven radiomics pipelines applied to bladder CT and multiparametric MRI have also shown promise for non-invasive staging and prediction of muscle invasiveness, with AUCs exceeding 0.90 in validation cohorts [39,40]. Integrative Exosome-SERS-AI platforms further illustrate how combining spectral exosomal profiles with deep-learning classifiers can detect early-stage cancers across multiple tumor types, including bladder, with sensitivities up to 85 % [69,70]. As datasets grow and algorithms become more transparent, AI will be critical for unlocking complex biomarker signatures from liquid biopsies [65,71].

### 7.3 Potential for novel targeted therapies

Molecular subtyping and liquid biopsy-driven target identification have enabled precision deployment of FGFR inhibitors, antibody-drug conjugates (ADCs) and other modalities. The pan-FGFR tyrosine kinase inhibitor erdafitinib received European Commission approval for *FGFR3*-altered unresectable/metastatic urothelial carcinoma, improving overall response rates to ∼32 % in heavily pretreated patients [28,72,73]. Ongoing trials such as SOGUG-NEOWIN are evaluating neoadjuvant erdafitinib regimens in high-risk NMIBC, while ctDNA-based FGFR testing is being compared against tissue assays to guide therapy selection [74,75]. Enfortumab vedotin, an ADC targeting nectin-4, in combination with pembrolizumab, has demonstrated objective response rates of 68 % and 1-year survival rates of 79 % in metastatic settings, establishing a new first-line standard for aUC [76,77]. Emerging agents - such as trastuzumab deruxtecan for HER2-positive tumors and novel *FGFR3*-centric tyrosine kinase inhibitors - are entering trials, guided by biomarker-driven eligibility and monitored via liquid biopsy for early signals of efficacy or resistance [[78], [79], [80]]. In summary, the integration of ctDNA-guided decision making, AI-enhanced biomarker interpretation and an expanding armamentarium of targeted therapies heralds a new era of truly personalized bladder cancer care [[81], [82], [83]]. Continued clinical validation, prospective trials and robust standardization will be essential to translate these innovations from bench to bedside.

## 8 Conclusions

Recent advances in urinary and blood-based assays - including methylation panels like Bladder EpiCheck, high-throughput CpG-targeting tests such as UroMark, multi-microRNA urine signatures, and plasma ctDNA detection - have significantly improved the sensitivity and specificity of bladder cancer detection and surveillance compared to conventional cytology and cystoscopy [[84], [85], [86]]. Molecular classification into luminal and basal subtypes, with further refinement into consensus classes, has enabled biologically informed therapeutic strategies - for example, FGFR inhibitors (erdafitinib) for luminal-papillary tumors and EGFR-targeted approaches for basal/squamous cases - ushering in a more precise treatment paradigm [[87], [88], [89]]. Emerging techniques in ctDNA-guided minimal residual disease monitoring and the application of artificial intelligence to integrate multi-omic liquid biopsy data promise to refine patient management by enabling dynamic treatment adaptation and early relapse detection [[90], [91], [92]]. Yet, clinical implementation is currently hampered by variability in pre-analytical procedures, lack of assay standardization, and a dearth of robust prospective validation and cost-effectiveness analyses - issues that must be addressed through harmonized protocols and large multicenter trials [93,94]. Looking ahead, integration of liquid biopsy with advanced imaging modalities, tissue biopsy, and digital pathology - supported by multidisciplinary collaborations, health-economic evaluations, and formal guideline endorsements - will be essential to realize the full potential of liquid biomarkers in personalizing bladder cancer care and improving patient outcomes [95,96].

## Ethical approval

This study did not involve experiments on human participants or animals conducted by any of the authors. All data discussed in this article were obtained from previously published studies, which had received appropriate ethical approval. For studies involving human subjects, all referenced works adhered to the ethical standards of the institutional and/or national research committee and the 1964 Helsinki Declaration and its later amendments or comparable ethical standards. In cases where patient data were utilized, they were anonymized to ensure confidentiality. No new clinical samples or patient-related interventions were performed specifically for this study. Therefore, ethical approval was not required.

## Declaration of competing interest

The authors declare that they have no known competing financial interests or personal relationships that could have appeared to influence the work reported in this paper.
